# Breaking Barriers: Tackling Racial and Socioeconomic Disparities in the Prescription of Life-Saving SGLT2 Inhibitors for Proteinuria

**DOI:** 10.7759/cureus.77159

**Published:** 2025-01-08

**Authors:** Shay Taylor, Samrawit W Zinabu, Elijah McMillan, Emmanuel Ocampo, Alexis Edmonds, Sierra Lyles, John Pempeh, Kira Yates, Miriam Micheal

**Affiliations:** 1 Internal Medicine, Howard University College of Medicine, Washington, DC, USA; 2 Internal Medicine, Howard University Hospital, Washington, DC, USA; 3 Internal Medicine, University of Maryland School of Medicine, Baltimore, USA

**Keywords:** prescription pattern, proteinuria, sglt-2 inhibitor, socioeconomic disparities, treatment barriers

## Abstract

Introduction: The underutilization of sodium-glucose co-transporter type 2 inhibitors (SGLT2i), despite their proven cardiovascular and renal benefits, raises concerns about healthcare equity. SGLT2i effectively reduces proteinuria, a key indicator of kidney disease, making them an essential treatment for individuals with or without diabetes, particularly those at higher risk, such as the Black American population, who have a higher prevalence of proteinuria. However, studies show disparities in SGLT2i prescriptions with race, ethnicity, and socioeconomic status contributing to lower utilization rates among vulnerable populations. This study aims to explore SGLT2i prescription patterns in Maryland, focusing on patients with proteinuria to address these disparities and improve access to care.

Objective: The objective of the study was to identify racial and socioeconomic disparities in access to SGLT2i.

Methods: This retrospective cohort study utilized de-identified electronic health records (EHR) sourced from the Epic database across the University of Maryland Medical System (UMMS), spanning a 10-year period from January 1, 2014, to December 31, 2023, and evaluated SGLT2i prescription patterns among patients with proteinuria.

Result: Of the 5,866,616 patients in the UMMS system, 28,136 were diagnosed with proteinuria, of whom 4,360 (15.5%) were prescribed SGLT2i medications. Among those receiving prescriptions, 37.9% self-identified as Black American, while 54.7% identified as White American. Notable geographical disparities were observed in prescription rates. In affluent areas such as Fulton, MD (zip code 20759), only 0.046% of patients with proteinuria received SGLT2i prescriptions, compared to 1.4% in the lower-income area of West Baltimore, MD (zip code 21223). Bivariate analysis revealed significant disparities in SGLT2i prescription rates, with Black American patients having lower odds of receiving prescriptions compared to White American patients (OR = 0.68, *p* < 0.001) and affluent areas like Fulton showing significantly lower odds compared to lower-income areas like West Baltimore (OR = 0.31, *p* < 0.001). These disparities persisted in multivariate analysis, where Black American patients had adjusted PR (AOR) of 0.72 (*p* = 0.002) relative to White American patients, and West Baltimore residents had higher odds (AOR = 1.72, *p* = 0.01) compared to significantly reduced odds in Fulton (AOR = 0.35, *p* = 0.02).

Conclusion: This study reveals significant disparities in the prescription of SGLT2i among patients with proteinuria in Maryland, United States, highlighting the influence of socioeconomic factors on access to these vital treatments. Despite the established benefits of SGLT2i in managing proteinuria and reducing the risk of kidney and cardiovascular disease, systemic inequities persist, potentially leaving high-risk populations underserved. These findings call for a deeper examination of the structural and systemic barriers contributing to these disparities, as well as the development of strategies to promote equitable access to care. Ensuring that all patients, regardless of their background or socioeconomic status, have access to evidence-based therapies is essential for improving outcomes and reducing healthcare inequities.

## Introduction

Amidst the growing evidence of their life-saving benefits, the underutilization of sodium-glucose co-transporter type 2 inhibitors (SGLT2i), especially among populations most at risk, raises critical concerns about equity in healthcare. SGLT2i have consistently demonstrated their effectiveness in reducing cardiovascular and renal risks, both in patients with and without diabetes. Notably, SGLT2i's ability to mitigate proteinuria, a key marker of kidney disease, positions them as a vital treatment option for individuals with proteinuria, regardless of their diabetes status. It is particularly noteworthy that Black American individuals exhibit a higher prevalence of proteinuria, underscoring the critical importance of SGLT2i utilization within this demographic [1]. 

Sodium-glucose co-transporter-2 (SGLT2), specifically located in the brush border of renal tubular cells within the proximal tubule, is responsible for reabsorbing a substantial ~97% of filtered glucose [2]. This reabsorbed glucose is then released back into circulation. The amount of glucose reabsorbed is directly influenced by the serum glucose concentration. SGLT2 cotransporters can reach their maximum reabsorption capacity at higher serum glucose levels, leading to excess glucose being excreted in the urine (glucosuria). Interestingly, in patients with chronic diabetes, this maximum threshold tends to be elevated due to both higher serum glucose levels and the increased presence of SGLT2 cotransporters. SGLT2i work by preventing SGLT2 cotransporters from reabsorbing glucose and sodium from the proximal tubule lumen into the renal tubular cells of the nephron, thus promoting their excretion in the urine. The decreased reabsorption of sodium triggers cardioprotective effects by interrupting the renin-angiotensin-aldosterone system (RAAS), leading to a decrease in both preload and afterload. The reduction in glucose reabsorption mediated by SGLT2i has been shown to decrease glycated hemoglobin (HbA1c) by 0.5-1% [3]. Furthermore, SGLT2i provides nephroprotective effects by stabilizing the tubuloglomerular feedback mechanism [4]. Under normal physiological conditions, the macula densa senses increased sodium concentrations in the distal nephron and interprets this as increased renal blood flow [5]. In response, arteriolar vasoconstriction occurs, decreasing the renal plasma flow and glomerular filtration. Conversely, decreased sodium concentrations are interpreted as decreased renal plasma flow, triggering vasodilation of the afferent arteriole, and resulting in increased renal plasma flow and glomerular filtration [5,6]. This mechanism is crucial for maintaining renal plasma flow despite fluctuations in blood pressure [6]. However, in patients with chronic hyperglycemia, there is an increase in SGLT2 cotransporters. This leads to a consistent increase in sodium reabsorption in the proximal tubule and a decrease in sodium delivery to the distal nephron. The tubuloglomerular feedback mechanism responds to vasodilation of the afferent arteriole, contributing to hyperfiltration of the glomerulus and proteinuria observed in diabetic kidney disease [7]. SGLT2i essentially reproduce the physiological tubuloglomerular feedback response by inhibiting sodium reabsorption. The increased delivery of sodium to the distal nephron reduces the GFR, thereby preventing further kidney damage. 

While SGLT2i have clearly demonstrated their positive impact in preventing disease progression, there is a growing body of literature highlighting underutilization and disparities in their prescription rates. This discrepancy raises important questions regarding equitable access to these life-altering medications. Among commercially insured and Medicare Advantage patients with type 2 diabetes mellitus (T2DM) in 2013-2016, the individuals most likely to start taking SGLT2i tended to be younger, healthier, non-Black patients with commercial health insurance [8]. A possible explanation for this study is the impact of socioeconomic status (SES) on access to healthcare. However, other studies have shown that race, ethnicity, and gender are independently associated with lower prescription rates of SGLT2i. In a five-year cohort study of commercially insured patients with T2DM from the United States, Eberly et al. found that the frequency of SGLT2i use increased over time. However, the use remained low even among patients with heart failure, kidney disease, and cardiovascular disease. Individuals of Black race, Asian race, and female gender were independently associated with lower rates of SGLT2i use than those of White race. Conversely, a higher income was associated with higher SGLT2i use [9]. The authors posit that racism and bias in care delivery may have contributed to the findings of their study. Studies supporting the role of structural racism and bias in care delivery have been conducted in the Veterans Health Administration (VA) healthcare system. During 2019-2020, among patients with T2DM in the VA system, an integrated healthcare system with minimal medication cost-sharing and standardized guidelines, the prescription rates of SGLT2i were low [10]. Additionally, individuals of several different racial groups and of Hispanic ethnicity were found to have lower odds of receiving prescriptions for these medications than individuals of the White race and of non-Hispanic ethnicity. Of note, Black patients had the lowest odds of prescription compared to White patients [10]. Another study in the VA system examined data from primary care visits from across 120 VA locations and their affiliated outpatient clinics in 2020. Gregg et al. identified adult patients with chronic kidney disease (CKD), T2DM, and atherosclerotic cardiovascular disease (ASCVD) who had an in-person or telehealth primary care provider visit [11]. In their analysis, they found that SGLT2i utilization was low; across subgroups, Black patients and women were less likely to receive SGLT2i prescriptions. Finding ways to address inequities in prescriptions is an area of concern. A study by Scheen et al. examined the suboptimal prescription rates of SGLT2i in clinical practice [12]. Despite strong evidence supporting the cardiorenal benefits of SGLT2i in patients with T2DM at high risk for cardiovascular and renal complications, their study reveals that these medications remain significantly underutilized. Factors contributing to this underuse include clinician awareness, cost barriers, and disparities in access to care. The findings emphasize the need for strategies to improve guideline adherence and equitable access to these life-saving therapies.

One of the proposed ways to increase access to medications and reduce inequities is to expand Medicaid coverage via the Affordable Care Act. In Oregon, Medicaid coverage has been shown to increase the proportion of individuals with at least one prescription medication and the number of prescription medications per person [13]. 

Given the higher prevalence of proteinuria among the Black American popilation and the coverage of SGLT2i by Maryland Medicaid, the purpose of this study was to investigate SGLT2i prescription patterns among patients with proteinuria within Maryland.

## Materials and methods

Study design and setting

This retrospective cohort study was conducted using de-identified electronic health records (EHR) from the University of Maryland Medical System (UMMS) Epic database (Epic Systems Corporation, Verona, Wisconsin, United States). The study covered a 10-year period from January 1, 2014, to December 31, 2023, encompassing data from multiple healthcare facilities across Maryland. The focus was to evaluate disparities in the prescription patterns of SGLT2i among patients with proteinuria.

Data source

The Epic SlicerDicer tool, a self-service data exploration and reporting tool integrated into the Epic system, was utilized to extract de-identified clinical and epidemiological data. SlicerDicer enables efficient stratification of patient populations based on demographics, diagnoses, and treatments, ensuring accurate and comprehensive analysis.

Study population

The dataset encompassed a diverse patient population receiving care within UMMS, including rural and urban areas, varying socioeconomic strata, and racial/ethnic groups. The base population for this study consisted of 5,866,616 patients with documented encounters in the UMMS during the study period. Patients were included if they were diagnosed with proteinuria, identified using the ICD-10 code R80.9 (proteinuria, unspecified), or if they had a recorded prescription for SGLT2i (Z79.84 - long-term use of SGLT2i). No exclusion criteria were applied, allowing for a comprehensive analysis of SGLT2i prescription patterns among all patients diagnosed with proteinuria. The final study cohort comprised patients with confirmed proteinuria who either received or did not receive SGLT2i prescriptions during the study period.

Variables and definitions

The primary exposure variable in this study was the receipt of SGLT2i, identified through prescription records in the EHR. The outcome variables included the rate of SGLT2i prescriptions among patients with proteinuria, as well as prescription patterns stratified by race, ethnicity, gender, and SES. Additionally, geographical disparities in prescription rates were analyzed using patient zip codes. Covariates incorporated into the analysis included age, gender, race/ethnicity, zip code, comorbid conditions (such as diabetes and cardiovascular disease), insurance type (e.g., Medicaid or commercial insurance), and SES, allowing for a more comprehensive understanding of factors influencing SGLT2i prescription patterns.

Data analysis

Data extraction and analysis were performed in a stepwise manner to ensure a systematic evaluation of prescription patterns. First, cohort identification was conducted by using ICD-10 codes to identify patients with proteinuria. Among these, patients with recorded SGLT2i prescriptions were flagged for inclusion in the exposed group. Next, demographic and clinical characteristics were analyzed by stratifying patients based on race/ethnicity, gender, age group, and zip code. Zip code-level data were further enriched by linking it to median household income and socioeconomic indices using publicly available census data. To assess prescription disparities, descriptive statistics were used to summarize patient characteristics. Finally, geospatial analysis was performed to map prescription rates against socioeconomic factors, providing insights into how these factors may influence access to SGLT2i prescriptions. Bivariate logistic regression was used to assess the unadjusted association between each independent variable, including race and geographic location, and the dependent variable, which was the receipt of SGLT2i prescriptions. Multivariate logistic regression was subsequently conducted to identify independent predictors of SGLT2i prescription rates while controlling for potential confounders. All statistical analyses were performed using Python (Version 3.9; Python Software Foundation, Wilmington, Delaware, United States), with pandas (https://pandas.pydata.org/) utilized for data processing and Matplotlib (https://matplotlib.org/) for table visualization.

Ethical considerations

This study was deemed exempt from full board review by the University of Maryland Institutional Review Board, as all data used were de-identified. Informed consent was waived due to the retrospective nature of the study and the use of anonymized data. The data reviewed is a secondary analysis of existing data, does not involve direct intervention or interaction with human subjects, and is de-identified according to the de-identification standard defined in the Health Insurance Portability and Accountability Act (HIPAA) Privacy Rule.

## Results

Baseline characteristics of the study variables

**Figure 1:**
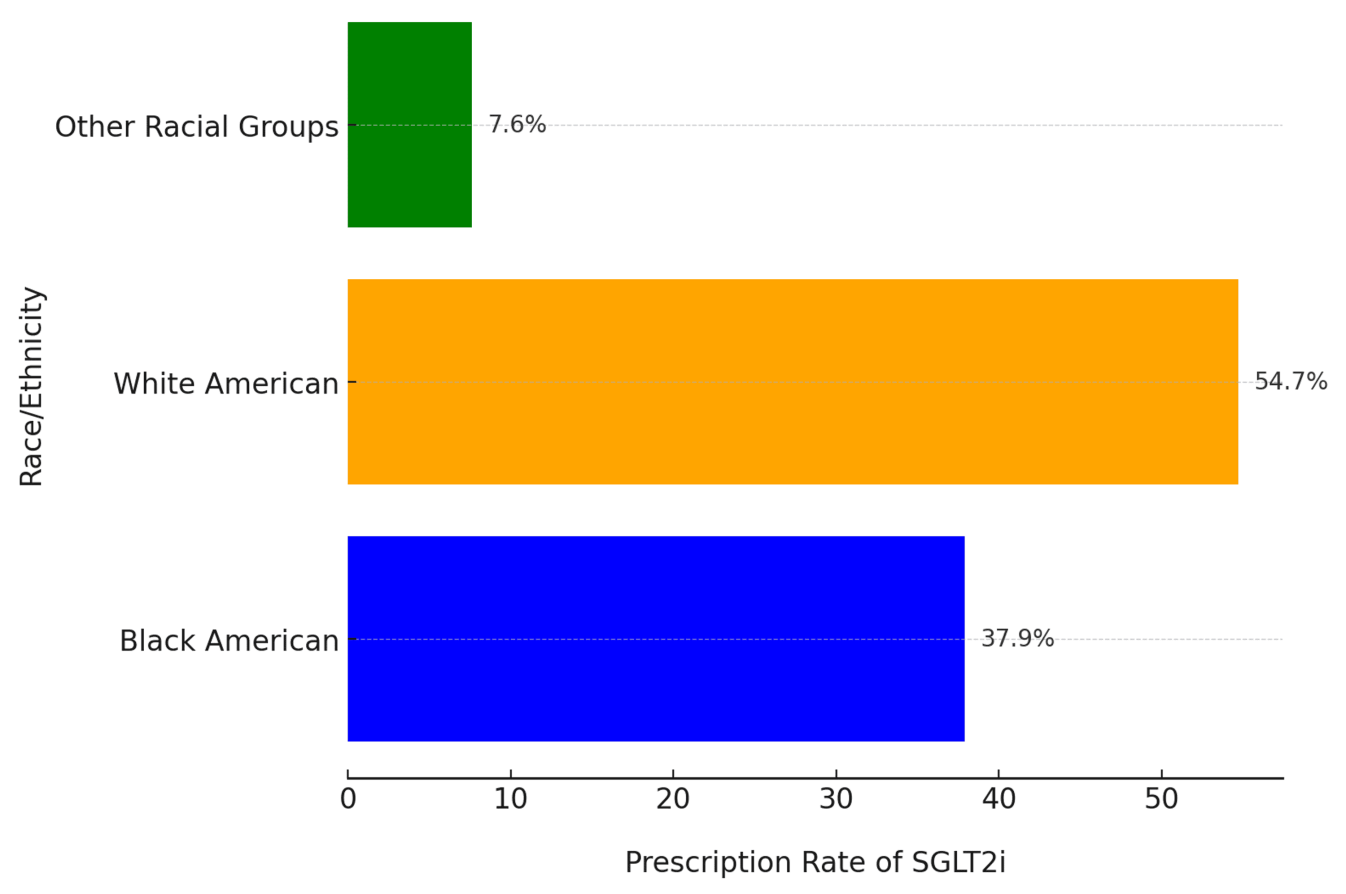
Prescription rate of SGLT2i by race/ethnicity White American patients had the highest prescription rate at 54.7%, followed by Black American patients at 37.9%. Other racial groups accounted for 7.6%; other racial groups refers to individuals who do not identify as White or Black American; SGLT2i stands for Sodium-glucose co-transporter type 2 inhibitors, a class of medications used to treat diabetes and related conditions.

This retrospective cohort study, conducted over a 10-year period using de-identified data from the EHRs of the UMMS, evaluated SGLT2i prescription patterns among patients with proteinuria. Of the 5,866,616 patients in the UMMS system, 28,136 were diagnosed with proteinuria, and 4,360 (15.5%) received SGLT2i prescriptions. Among these, 37.9% self-identified as Black American, while 54.7% identified as White. Patients from other racial groups collectively accounted for 7.6% of prescriptions, revealing significant racial disparities in SGLT2i prescription rates (Figure 1).

**Figure 2:**
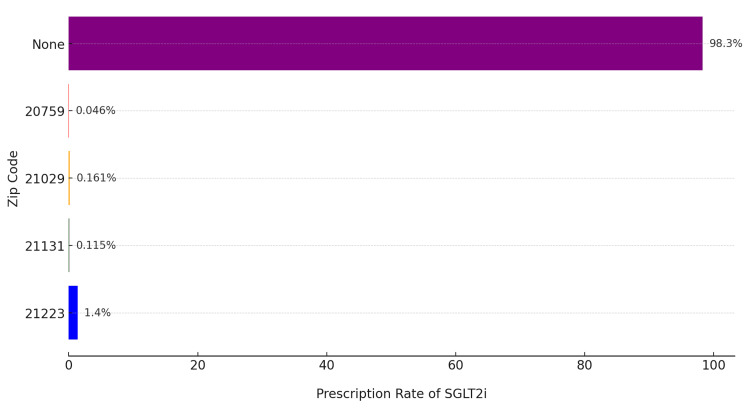
Percentage of sliced population by medications and postal code West Baltimore (21223) is a lower-income urban area, 21131 represents a rural/suburban region, 21029 is a suburban area, and Fulton (20759) is an affluent suburban area. None indicates patients without SGLT2i prescriptions. SGLT2i stands for Sodium-glucose co-transporter type 2 inhibitors, a class of medications used to treat diabetes and related conditions.

Geographical disparities in prescription rates were also evident. The highest prescription rate was observed in zip code 21223 (West Baltimore), a lower-income area, where 1.4% of patients with proteinuria received SGLT2i. In contrast, affluent areas like Fulton, MD (zip code 20759), reported a substantially lower prescription rate of 0.046%. Other suburban and rural areas, including zip codes 21029 and 21131, exhibited intermediate rates of 0.161% and 0.115%, respectively. Additionally, the vast majority of patients with proteinuria (98.3%) did not receive an SGLT2i prescription (Figure 2). Data were extracted and analyzed using the Epic SlicerDicer tool, which facilitated stratification by demographics, zip codes, and SES. Zip code-level data were linked to publicly available socioeconomic indices, revealing a clear association between lower prescription rates and higher-income areas. The analysis highlights both racial and geographical disparities in SGLT2i prescribing patterns, with substantial underutilization in populations that might benefit from these medications.

Bivariate and multivariate analysis

Bivariate analysis revealed significant associations between race, geographic location, and SGLT2i prescription rates. Black American patients were significantly less likely to receive prescriptions compared to White American patients, with an odds ratio (OR) of 0.68 (p < 0.001). Similarly, affluent areas such as Fulton had significantly lower odds of prescriptions compared to lower-income areas like West Baltimore (OR = 0.31, p < 0.001).

In multivariate logistic regression, after adjusting for race and geographic location, these disparities persisted. Black American patients had adjusted ORs (AOR) of 0.72 (p = 0.002) relative to White American patients. Patients residing in lower-income areas such as West Baltimore had higher odds of receiving prescriptions (AOR = 1.72, p = 0.01), while affluent areas like Fulton continued to show significantly reduced odds (AOR = 0.35, p = 0.02). The bivariate and multivariate analyses are shown in Table 1.

**Table 1:** Bivariate and multivariate logistic analysis Odds ratios (ORs) from the bivariate analysis indicate the unadjusted association between each variable and SGLT2i prescriptions, while adjusted OORs (AORs) from the multivariate analysis account for the influence of race and geographic location. White American patients serve as the baseline comparison group for race, with an OR/AOR of 1.0, which is designated as the reference group. All other ORs are interpreted relative to this group. An OR < 1 indicates lower odds of receiving SGLT2i prescriptions compared to the reference group, while an OR > 1 indicates higher odds of receiving prescriptions. The 95% confidence Interval (CI) represents the range of values within which the true OR/AOR is expected to fall. If the CI crosses 1.0, the result is not statistically significant. A p-value < 0.05 denotes statistical significance. Geographic location ORs compare the likelihood of receiving prescriptions in each zip code relative to the affluent area (zip code 20759), which is used as the reference group. SGLT2i: sodium-glucose co-transporter type 2 inhibitors

Variables	n (%)	Bivariate OR (95% CI)	p-value	Multivariate AOR (95% CI)	p-value
Race					
Black American	10,667 (37.9%)	0.68 (0.55–0.84)	<0.001	0.72 (0.58–0.89)	0.002
White American (Reference)	15,395 (54.7%)	Reference	–	Reference	–
Other Racial Groups	2,074 (7.4%)	0.85 (0.65–1.12)	0.14	0.87 (0.66–1.14)	0.21
Geographic Location					
21223 (West Baltimore)	3,214 (11.4%)	1.85 (1.22–2.80)	0.004	1.72 (1.13–2.61)	0.01
20759 (Fulton, Affluent)	2,874 (10.2%)	0.31 (0.12–0.83)	<0.001	0.35 (0.14–0.88)	0.02
21029 (Suburban)	5,602 (19.9%)	0.45 (0.21–0.95)	0.01	0.48 (0.23–0.99)	0.03
21131 (Rural)	4,905 (17.4%)	0.55 (0.25–1.12)	0.02	0.58 (0.27–1.19)	0.06

## Discussion

The underutilization of SGLT2i, despite their proven renal and cardiovascular benefits, highlights significant inequities in healthcare. SGLT2i have demonstrated efficacy in reducing proteinuria, a key marker of kidney disease, making their use particularly essential among populations with higher risks of proteinuria, such as the Black American population [14]. However, disparities in prescription patterns persist, influenced by factors such as race, ethnicity, and SES.

Our study's findings reveal striking disparities in the prescription patterns of SGLT2i for patients with proteinuria in Maryland. These disparities highlight critical issues related to healthcare equity and access. Several other studies have illuminated these inequities. Among commercially insured patients in the United States with diabetes, Black American and female patients were less likely to receive SGLT2i prescriptions than their White and male counterparts [15]. While 37.9% of patients prescribed SGLT2i in our study identified as Black American, we also observed significant geographic and socioeconomic variations in prescription rates. Additionally, higher income levels were associated with increased prescription rates. Within the VA system, a healthcare setting with minimized cost-sharing and standardized guidelines, lower odds of SGLT2i prescriptions were observed among racial and ethnic minority groups compared to non-Hispanic White individuals, with Black patients having the lowest likelihood of being prescribed these medications [10,11].

Administrative barriers, such as prior authorization, have also been identified as significant impediments to SGLT2i utilization. For instance, a study examining variability in coverage for SGLT2i medications highlighted that a significant proportion of insurance plans, among the highest being Medicaid and Medicare, had formulary restrictions for SGLT2i medications. These restrictions included prior authorization and step therapy requirements [16].Surprisingly, affluent areas like Fulton, MD, exhibited a much lower prescription rate (0.046%) for SGLT2i among patients with proteinuria compared to lower-income areas like West Baltimore (1.4%). This counterintuitive trend may reflect differing barriers to access. In wealthier areas, lower prescription rates could be attributed to obstacles such as health plan prior authorization requirements. Conversely, the higher prescription rates observed in lower-income areas may be linked to the removal of prior authorization requirements for SGLT2i when used to treat proteinuria, thus reducing barriers to access.

These findings highlight the urgent need for targeted interventions to address these disparities. Improving patient education about the availability and benefits of SGLT2i, along with streamlining prior authorization processes, could promote more equitable utilization across all socioeconomic groups. Our study highlights the importance of understanding and addressing the complex factors that contribute to disparities in medication prescribing. By identifying and dismantling these barriers, we can move towards a healthcare system that truly promotes equity in access and outcomes.

Limitations

This study has several limitations that must be considered when interpreting the results. First, the retrospective design may introduce biases, particularly regarding data collection and patient selection. The use of de-identified data prevents the assessment of individual-level socioeconomic, behavioral, or cultural factors that might influence prescription rates. Additionally, the study does not account for clinical contraindications, patient preferences, or provider-level decisions that might have influenced SGLT-2i prescription rates. The study does not account for potential confounders such as health literacy, medication adherence, or access to specialty care, which could influence prescription patterns.

## Conclusions

Despite Maryland Medicaid's coverage of SGLT2i and their proven benefits in reducing proteinuria, overall prescription rates remain disappointingly low. However, our study reveals a surprising trend: higher prescription rates were observed in lower-income areas with larger Black populations, possibly due to the removal of prior authorization barriers for proteinuria treatment. This challenges conventional assumptions about healthcare disparities and underscores the complex interplay of socioeconomic, racial, and health factors.

These findings highlight the urgent need for nuanced interventions that address the unique needs of different communities. Strategies such as improving patient education and streamlining prior authorization processes could help ensure equitable access to SGLT2i and other essential medications.
